# Supplementary material for: A Global Screen for Assembly State Changes of the Mitotic Proteome by SEC-SWATH-MS
Source: Cell Syst. 2020 Feb 26;10(2):133–155.e6. doi: 10.1016/j.cels.2020.01.001 (PMC7042714; doi:10.1016/j.cels.2020.01.001)

Q9GZY8 | MFF_HUMAN | MFF C2orf33 AD030 AD033 GL004
Monomer MW [kDa]: 38.465 Monomer expected elution fraction: 49

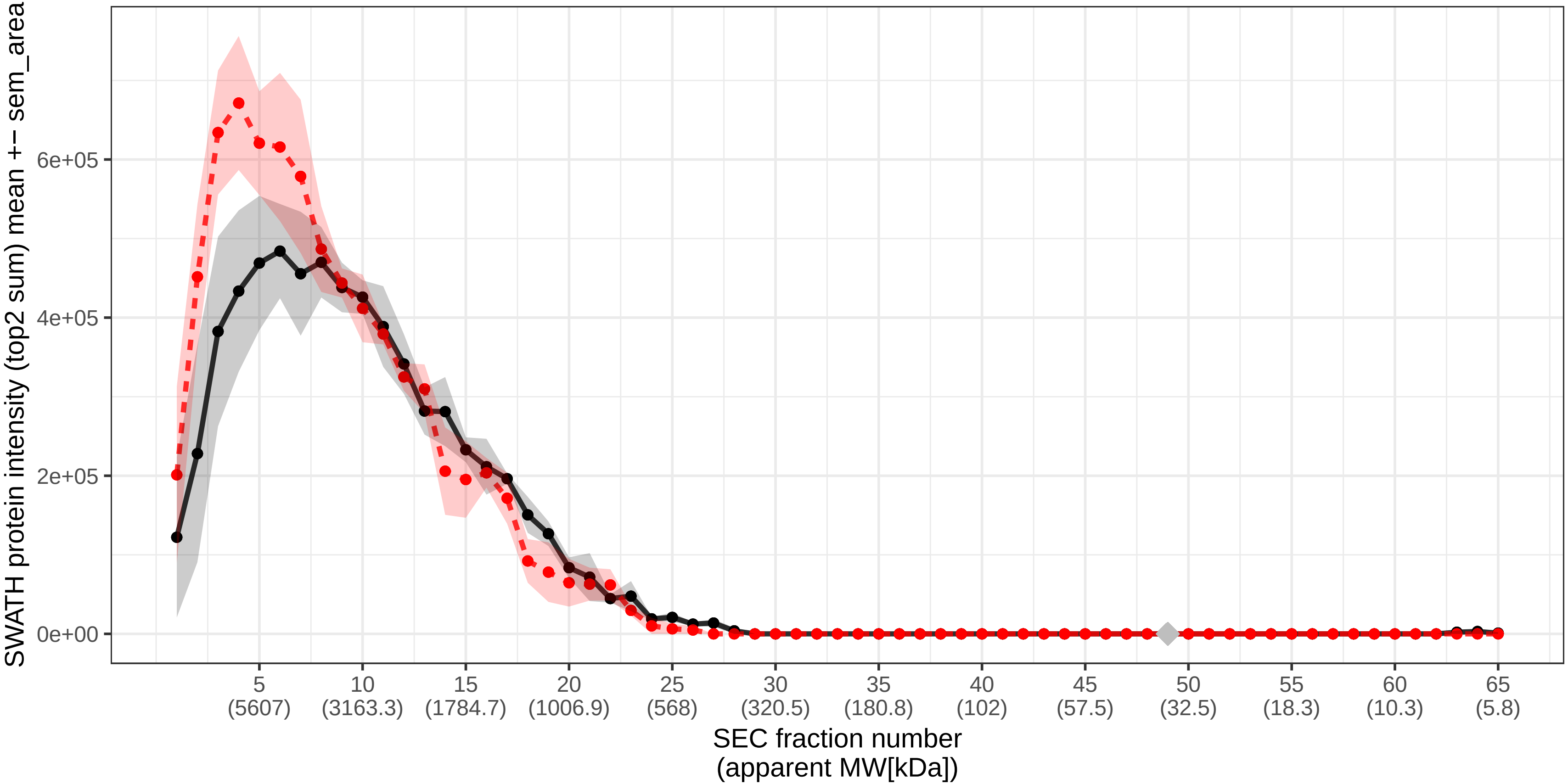

Supplement: Data S1. SEC-SWATH-MS Protein Chromatograms, Related to Figure 1 [file mmc6.zip › SECchrom_Q9GZY8_MFF_HUMAN_MFF_C2orf33_AD030_AD033_GL004.pdf]
